# Clinical applications of circulating tumor cells in patients with solid tumors

**DOI:** 10.1007/s10585-024-10267-5

**Published:** 2024-01-28

**Authors:** Daniel J. Smit, Svenja Schneegans, Klaus Pantel

**Affiliations:** 1 Institute of Tumor Biology, University Medical Center Hamburg-Eppendorf, Martinistraße 52, 20246 Hamburg, Germany; 2 Fleur Hiege Center for Skin Cancer Research, Institute of Tumor Biology, University Medical Center Hamburg-Eppendorf, Martinistraße 52, 20246 Hamburg, Germany

**Keywords:** Liquid biopsy, Circulating tumor cells, Clinical application, Precision medicine

## Abstract

The concept of liquid biopsy analysis has been established more than a decade ago. Since the establishment of the term, tremendous advances have been achieved and plenty of methods as well as analytes have been investigated in basic research as well in clinical trials. Liquid biopsy refers to a body fluid-based biopsy that is minimal-invasive, and most importantly, allows dense monitoring of tumor responses by sequential blood sampling. Blood is the most important analyte for liquid biopsy analyses, providing an easily accessible source for a plethora of cells, cell-derived products, free nucleic acids, proteins as well as vesicles. More than 12,000 publications are listed in PubMed as of today including the term liquid biopsy. In this manuscript, we critically review the current implications of liquid biopsy, with special focus on circulating tumor cells, and describe the hurdles that need to be addressed before liquid biopsy can be implemented in clinical standard of care guidelines.

## Introduction

Despite tremendous efforts in clinical and basic cancer research, metastasis remains a key driver of cancer-associated mortality [1]. Therefore, early detection of both localized as well as metastasized cancers is of utmost importance for the survival of these patients. During metastasis, cancer cells detach from the primary tumor, infiltrate the local tissue, intravasate into blood vessels and ultimately invade into distant organs, where they need to be able to proliferate to form new metastases [2]. To achieve this, the tumor cells need to switch from an immotile to a more migratory state, a process called epithelial–mesenchymal transition (EMT), during which epithelial molecules are downregulated while simultaneously mesenchymal proteins are upregulated [3]. Moreover, EMT leads to the activation of several transcription factors such as Slug, Snail, Twist, and ZEB1/2 [3], ultimately resulting in alterations in signalling pathways changing polarity, cell-cell contacts and extracellular matrix composition, therefore enhancing invasive properties [4]. Once the detached cells arrive at the metastatic site, these traits need to be reversed through MET (mesenchymal to epithelial transition) to facilitate re-attachment, proliferation, and metastatic outgrowth [4].

Tumor cells that have undergone this process are called disseminated tumor cells (DTCs) and are frequently found in the bone marrow or lymph nodes. Other major, clinically relevant sites of metastases include the liver, the lung, the bone and the brain but are dependent on the anatomically determined lymphatic and venous drainage, the molecular characteristics of the primary tumor as well as the corresponding microenvironments of the secondary organs [5]. The presence of DTCs in the bone marrow negatively correlates with the survival of patients with various cancer entities [6, 7]. DTCs are tumor cells that have left the lymphatic or blood vessels and subsequently settled at lymph nodes or distant sites (e.g., bone marrow). DTCs occur at very low concentrations (e.g., 1 per 1 million bone marrow cells and highly sensitive methods are required for their detection (e.g., immunocytochemistry with keratin antibodies). Considering the total mass of bone marrow, it can be estimated that an early stage breast cancer patient at initial diagnosis can harbor hundred thousands of DTCs in the bone marrow and approximately 50% of these patients can control this tumor load over 10 years [8]. Understanding the mechanisms of this remarkable control might open new avenues for therapeutic interception aimed to block the development into full blown and incurable metastasis. To date, we know that DTCs can adapt to different microenvironments, reside in a non-proliferate (Ki67-negative) state, express oncogenes such as HER2, develop resistance against systemic anti-cancer treatment as well as acquire cancer stem cell (CSC)-like traits [9–11]. Bone marrow aspiration is an invasive procedure routinely used in patients with haematological malignancies but not in patients with solid tumors. Despite the promising clinical research studies on DTCs, the emerging interest in blood analyses (e.g., CTCs and ctDNA) has lowered the interest to implement DTC analyses into clinical protocols. However, we are confident that DTC analyses would add an important information that might be not obtained by minimally invasive blood tests which provide only a snap shot. Tumor cells that can be detected in the circulation are called circulating tumor cells (CTCs), however, as opposed to DTCs, they have not settled yet in a distant organ. For survival, these cells that have detached from the primary tumor or secondary metastatic sites circulate in the blood stream and have to withstand extreme conditions including high pressure within the vessels as well as evade immune surveillance mechanisms and adapt to a new microenvironment [12]. Unlike tissue biopsies, sampling of CTCs not only allows a comprehensive assessment of the state of the disease but also represents a non-invasive tool for sequential and real-time monitoring of tumor burden in cancer patients. Blood as a liquid biopsy source provides a convenient, easily accessible, and fast way of sampling and enables the investigation of different analytes apart from CTCs such as circulating nucleic acids (ctDNA, miRNAs), extracellular vesicles (EVs) and platelets. All analytes can provide complementary information on the (epi-)genomic, transcriptomic, and proteomic level which are highly valuable for the molecular characterization of the tumor burden in an individual cancer patient [13].

## Analysis of CTCs to understand the biology of metastasis

While in early-stage cancer the primary tumor serves as the main source for CTCs, ctDNA and EVs (and in later stages also the lymph nodes), in more advanced cancers, metastases are mainly responsible for releasing CTCs and other cancer cell derived products into the circulation [14]. Using CTCs as liquid biomarkers presents a challenge, not only because of their short half-life of approximately 1-2.4 h [15] but also because especially in early stages the number of CTCs shed into the peripheral blood circulation is low compared to the vast background of blood cells. It is estimated that one CTC can be found per approximately 10^6^-10^8^ leukocytes [16], however, this number also strongly varies between the different tumor entities and disease stages. For analysis of CTCs, enrichment is thus indispensable and can be performed using a variety of devices and techniques.

By making use of the physical and mechanical properties of CTCs and leukocytes, separation of these two cell populations can for example be achieved through differences in size, deformability, density, or electric charges using devices, with new technologies being constantly developed [17].

Biological properties such as expression of specific cell surface markers on the other hand, are furthermore necessary to ultimately distinguish between CTCs and leukocytes and thus for their identification and enumeration and therefore prognostic and diagnostic value.

The most prominent CTC marker EpCAM (epithelial cell adhesion molecule) is currently being utilized in the only FDA-approved CTC detection platform to date, the CellSearch system, in which CTCs are enriched through adhesion to anti-EpCAM-conjugated magnetic beads. However, during metastasis, cells detaching from the tumor sites and undergoing EMT, often show a significant downregulation of epithelial proteins such as EpCAM [18]. Negative depletion of immune cells including anti-CD45 conjugates for leucocyte depletion is therefore another option of CTC enrichment. The epithelial ImmunoSPOT (EPISPOT) assays can be used to detect cancer-specific proteins secreted by viable CTCs such as cytokeratins, having proven to show prognostic value in breast, colon, prostate, and head and neck tumors and melanoma [17, 19]. Moreover, the EPIDROP assay is currently being developed for detection of CTCs in microdroplets at the single cell level [20].

**Fig. 1:**
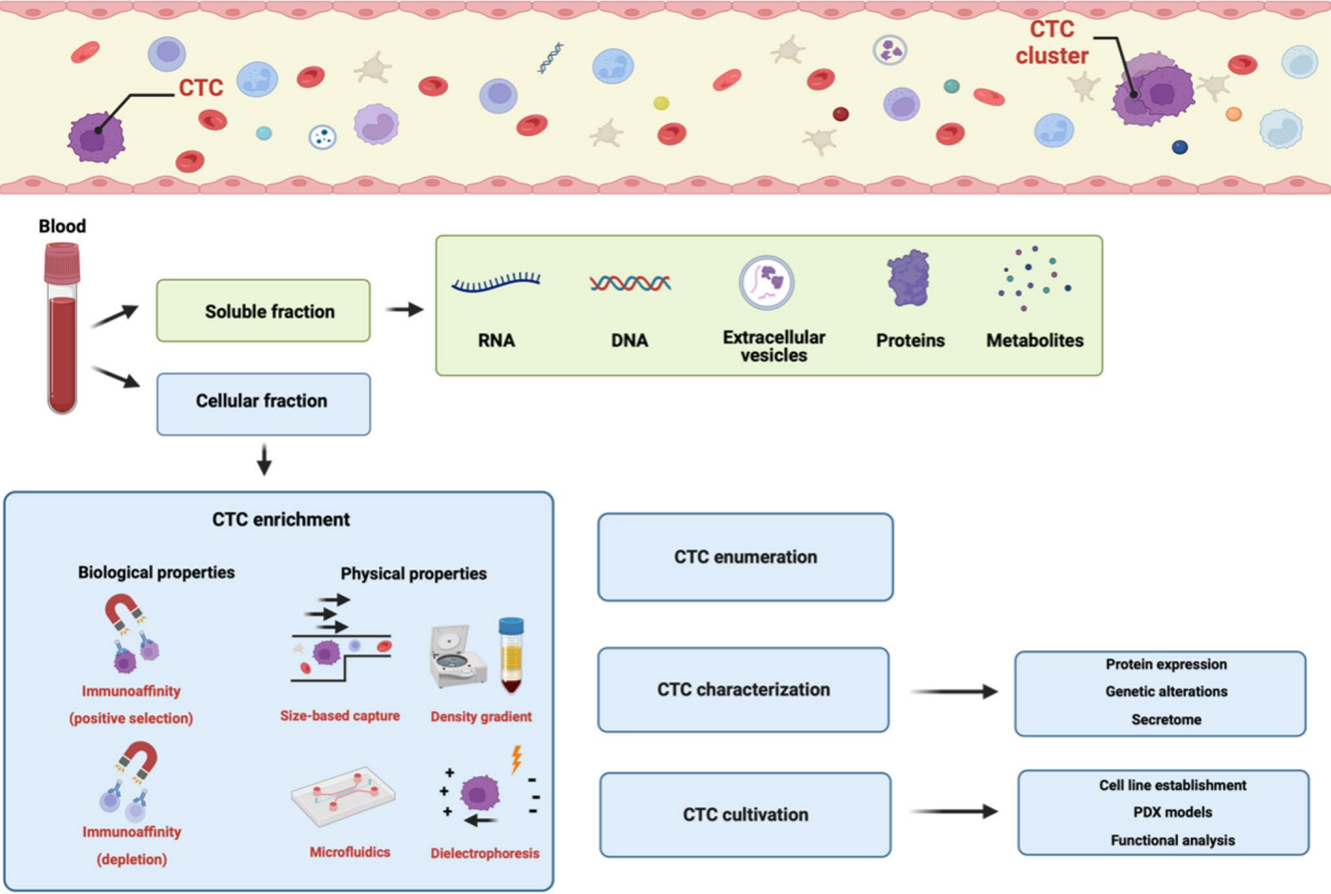
Overview of analytes in blood-based liquid biopsy analysis and CTC detection. Body fluids represent a rich source of cells as well as acellular products that can be assessed by liquid biopsy. Although liquid biopsy can be applied to all body fluids, the blood is most popular fluid within the field. Blood is composed of a cellular as well as acellular fraction. Within the soluble fraction, several liquid biopsy analytes including cell-free RNA, cell-free DNA, extracellular vesicles, (micro-)proteins as well as metabolites can be isolated. Circulating tumor cells (CTCs), if present, are surrounded by a plethora of other cells within the cellular fraction [14]. In order to isolate the CTCs from the blood sample, enrichment is required due to their low abundance. Several methods for CTC enrichment have been described in the past utilizing either biological properties of CTCs (in positive enrichment) such as marker expression or surrounding cells (depletion / negative enrichment) including immune cells. In addition to marker-based strategies, label-free methods based on physical properties have been established that allow the enrichment regardless of their marker expression for unbiased CTC detection. The enriched CTC fraction can be used for CTC enumeration but also for CTC molecular characterization as well as CTC cultivation for further insights into CTC biology [17, 19, 21]

Through these various techniques, an enrichment of CTCs by several log scales can be achieved which is necessary for effective subsequent molecular characterization on a single cell level [20] (Fig. 1).

 For identification of CTCs, immunocytochemistry is often used to detect population-specific (e.g. EpCAM, cytokeratins, CD45), tissue- or tumor-specific or EMT-related antigen expression [17]. For a more extensive characterization, however, down-stream methods which allow for RNA or DNA analysis through e.g. RT-qPCR, RNAseq, whole genome amplification (WGA) and subsequent next generation sequencing (NGS) and/or proteomic analyses are needed to fully comprehend the state of the tumor. The detection of CTCs represents a highly specific blood-based technique that could be utilized for early detection strategies. However, as the number of CTCs is very low and enrichment procedures (negative, as well as positive enrichment) potentially lead to a loss of CTCs, the use of CTCs for early detection may lack sensitivity and negative predictive value. While positive enrichment methods based on cancer entities or primary tumor characteristics may provide a strong enrichment of CTCs, studies have also demonstrated a high degree of heterogeneity and discordance in primary tumor characteristics and detected CTCs. To increase the yield of CTCs, the use of larger blood volumes or diagnostic leukapheresis products (DLA) has been suggested. In metastatic breast cancer, an increase of almost 21-fold was observed by the authors comparing the CTC counts in peripheral blood with DLA products using the CellSearch platform. Strikingly, in two patients that had no detectable CTCs in the peripheral blood, CTCs were detected in the DLA products underlining the increase in sensitivity [22].

 To further explore the functional properties of CTCs, suitable experimental models are needed. One good example for this is the establishment of in vitro CTC cell lines from the blood, even though being quite challenging. These model systems can be used to help understand the biology of CTCs, such as their heterogeneity in morphology, EMT status and features relevant for the development of metastasis [23]. Furthermore, more patient-relevant knowledge can be obtained, such as the (druggable) mutational status of both primary tumor and metastasis, evolutionary events, as well as potential drug sensitivities and resistances [23, 24].

 In most studies, the average blood volume needed for CTC cell line establishment varies between 6 and 18 mL. Even though higher CTC counts from the same patient tend to have a higher success rate, which is often only the case in advanced stages, this property alone does not guarantee a successful establishment [23, 24]. For example, during the establishment of the first long-term stable CRC cell line, out of 71 patients only 2 patients (with 302 and 516 CTCs in their sample) gave rise to an CTC line ex vivo, of which one was only stable two months, whereas the latter was successfully established [25]. Nevertheless, Brungs et al. reported the establishment of a CTC cell line (UWG01CTC) from a distal oesophageal high grade neuroendocrine carcinoma patient with only 3 detectable CTCs (per 7.5 mL blood) [26]. In general, the success rates are relatively low, requiring several patient samples before successful establishment of a long-term stable CTC cell line [23, 27]. However, once established, CTC cell lines can serve as an excellent tool for drug screening applications and thus have the potential to become highly valuable in personalized medicine [27]. The use of DLA products can increase the yield and thereby the success rate of establishing an ex vivo CTC culture as described earlier by Mout et al. that reached a success rate of 35% for organoid expansion [28].

 In contrast to commercially available, immortalized cell lines established from primary tumor tissue and metastatic sites, only a few CTC cell lines are available. In 2021, more than 300 successfully established CTC cell cultures have been reported, however, only less than 50 of these were stable for more than 6 months in culture [27]. Long-term stable CTC lines and also short-term cultivation of patient CTCs allow the analysis of viable cells and thereby pave the way towards a better understanding the biology of CTCs and identification of druggable targets.

 The first permanent cell line derived from colon cancer CTCs (CTC-MCC-41) was successfully established by Cayrefourcq et al., which proved to harbor similar characteristics as the primary tumor [25]. The cell line was found to be highly sensitive towards AKT and mTOR inhibition, which suggests a possible benefit for the patient using targeted therapy approaches [29]. Moreover, in 2020, Koch et al. established a CTC cell line (CTC-ITB-01) from an ER^+^ breast cancer patient which showed high concordance between their primary CTCs. Furthermore, its CNA (copy number alteration) profile remains stable during culture. Of note, drug sensitivity testing of CTC-ITB-01 cells, also revealed a high susceptibility for CDK4/6 inhibitors, that was not administered to the patient who gave rise to the cell line and may represented a value treatment option [30]. Another noteworthy example of a long-term CTC cell line (> 2 years in culture) is the CTC-TJH-01 line, a non-small cell lung cancer CTC line, established by Que et al. [31]. The cell line exhibited an intermediate epithelial-mesenchymal phenotype, stem cell-like traits as well as an increased immune escape compared to established lung cancer cell lines including A549 [31]. Functionally, the authors demonstrated the tumorigenic and metastatic capacity in a murine xenotransplantation model in vivo after subcutaneous as well as intravenous (tail vein) injection. In line with the reported concordance of the primary tumor and CTCs in the CTC-ITB-01 cell line, the CTC-TJH-01 cell line shared wild type EGFR and a missense mutation at codon 12 of KRAS with the primary tumor [31].

 These models thus prove to have a high prognostic value; however, their establishment remains challenging and is therefore only feasible in few selected patients. Using in vivo models, patient-derived xenografts (PDX) are furthermore suitable for the investigation of the potential to form metastases through injection of CTCs into immunodeficient mice. After intraductal injection of the CTC-ITB-01 cell line established by Koch et al., primary tumor growth was observed as well as spontaneous bone metastases, mirroring clinically relevant metastatic sites in ER^+^ breast cancer [30]. Consistently, Yu et al. reported the establishment of breast cancer CTC lines that retained ER positivity in 5 out of 6 lines. Moreover, in a murine subcutaneous xenotransplantation three CTC lines demonstrated tumorgenicity within 3 months with a matching molecular profile with regard to the respective primary tumor [32]. Similarly, the CTC-MCC-41 cell line as well as the corresponding primary tumor and xenografts harbor the same *KRAS* and *BRAF* mutations as well as CK20 expression [25]. The high capacity to retain the original molecular traits has also been confirmed in lung cancer, as demonstrated by CTC derived explants that were harvested after injection of CTCs into immunodeficient mice [33]. Faugeroux et al. established and characterized a prostate CTC-derived eXplant (CDX) model of model of neuroendocrine transdifferentiation in human castration resistant prostate cancer that allowed to study genetic and epigenetic mechanisms to further understand the biology of transdifferentiation and identify novel drug targets [34]. Overall, PDX models have shown metastasis formation in relevant organs and matching patient history which can be predictors of tumor development, ultimately representing a valuable tool in personalized medicine.

## Circulating tumor cells in the clinical setting

 The prognostic value of CTC detection has been extensively characterized in the past in different tumor entities. Detectable CTCs at the time of diagnosis are associated with reduced progression free and overall survival in many solid cancer entities [35–37]. In particular in breast cancer, there are numerous large-scale studies demonstrating a prognostic role of CTCs [38–41]. Similarly, for advanced/metastatic cancers or in relapse a negative prognosis has been already established in the past [42]. Although CTCs play a strong prognostic role in late stage cancers, the perspective of the use of CTC detection for early detection of cancer has been recently highlighted [43].

 Molecular characterization of CTCs can also provide information on therapeutic targets. One example are the DETECT-III trials, the first randomized interventional study indicating clinical utility of CTC characterization that revealed that breast cancer patients with HER2 negative tumors have HER2 positive CTCs in the circulation. Strikingly, it has been demonstrated that these patients benefit from anti-HER2/anti-EGFR treatment using Lapatinib with regard to increased overall survival rates [44–47]. Although the reported CTC positivity rate of nearly two third [44] is consistent with other studies in metastatic breast cancer due to the EpCAM-based enrichment of the CellSearch system, EpCAM^low^ or EpCAM negative CTCs may be lost [48]. False-negative results for CTC positivity may be therefore reduced in the future by using additional CTC markers, label-free approaches, DLA products or in vivo capture systems [14, 49].

 Despite the discordance of HER2 negative primary tumors and HER2 positive CTC reported in breast cancer, in lung cancer, a recent study analyzed the PD-L1 expression on CTCs, cytology imprints and tissue samples of NSCLC patients. The authors concluded that in clinical situations in which no tissue is available, the combination of cytological imprints and PD-L1 positive CTCs in the blood provides a predictive tool for tumoral PD-L1 status in NSCLC patients [50]. Moreover, a study on ALCAM expression in NSCLC patients with brain metastasis demonstrated that despite the high degree of heterogeneity of ALCAM expression on CTCs, a high concordance between ALCAM expression on CTCs and matched brain metastasis of the same patient was present [51].

 For treatment monitoring, a meta-analysis also reported that in breast cancer patients CTC counts decrease after anti-tumor therapy and can therefore be used as an indicator of treatment response [52]. This may implicate the use of CTCs as a prognostic marker in the clinical routine or even implicate the ability to make use of the CTC counts as a real time marker for monitoring response rate while undergoing anti-tumor therapy.

## Combination of liquid biopsy analytes

 Due to the complementary nature of the different analytes found in the blood, numerous studies have demonstrated the great potential of combining CTC- and further liquid biomarker analyses for diagnostic and prognostic purpose [53]. However, the information obtained in these multiparameter studies greatly depends on the type of analyses being performed. Most studies use enumeration of CTCs which has been a marker for progression- and disease-free survival in singular studies of various cancer entities for over a decade and combined it with ctDNA detection which proved to enhance sensitivity and specificity [54–57].

On the other hand, analysis of genomic CTC DNA as a complementary analyte can give rise to a deeper understanding of the mutational state of the tumor cell and can thus provide insight into its spatial and temporal heterogeneity. Combined with ctDNA analysis, mutational profiling of both analytes was previously performed in several studies [58–60] and was found to reveal the same mutations in breast cancer patients [61, 62]. However, also mutations exclusively found in either one of the analytes were identified [61, 62]. Therefore, analysis of both sample types complements each other so that additional information can be obtained. Due to the diverse nature of analytes in the blood, the possibility of combining liquid analytes in not limited and thus, combined analysis of CTC with EVs (including their cargo), cell- free RNAs, circulating proteins, DNA methylation, platelets, as well as other cell types found in the circulation are all accounted in ongoing research and can add complimentary value [12, 63, 64].

 A recent example of the combination of CTCs and tumor-derived EVs (tdEVs) has been published by Nanou et al. The authors examined the presence of tdEVs in both arms of the SWOG0500 trial (CTC response and non-response after the first treatment cycle). Nanou et al. demonstrated that the enumeration of tdEVs provides a strong prognostic marker complementary to the CTC response to further stratify patients into different risk groups [65].

 Moreover, the feasibility of detecting more than two liquid analytes has already been successfully proven, however, pre-analytical parameters such as the choice of blood collection tube, and time to processing, among other factors, should be carefully taken into consideration [63, 66]. The combination of CTCs, ctDNA, EVs, proteins and metabolites is currently being assessed in the EU-funded PANCAID consortium [67] with the goal to detect pancreatic adenocarcinomas earlier than current imaging tools.

## Standardization of liquid biopsy analysis and current clinical recommendations

 Since the discovery of liquid biopsy as a tool for cancer diagnostics and prognostics over a decade ago [68], a multitude of isolation and analysis techniques have been developed. Furthermore, novel biomarkers such as CAFs, or CECs that can be found in the blood circulation are the topic of ongoing investigations and help to characterize the tumor (and its tumor microenvironment) more precisely, providing a deeper understanding on the state of the disease, and possible treatment options.

 However, these advances also add a new level of complexity to clinical decision making. For a successful implementation into clinical practice, numerous pre-analytical and analytical factors must be taken into consideration [69]. Parameters such as sampling method and consumables, storage time, storage temperature, and moreover patient medication, time of sampling and comorbidities can all have an influence on the liquid analytes [66, 70]. Furthermore, a deep understanding of the correct choice of analyte and assay is urgently required to obtain and interpret the information suited for the respective patient and cancer entity. This multitude of parameters contributes to the lack of standardization and represents the biggest hurdle on the way to clinical implementation, when in fact, clinical practice needs guidelines and needs to be reliable and comparable.

 To this day, numerous CTC detection methods are available and are used in research studies. Several efforts have been made to evaluate these methods to assess their technical validity and enhance their applicability in a clinical setting [71, 72]. Koch et al. demonstrated the necessity of studying pre-analytical and analytical parameters, and furthermore defining optimal conditions for CTC enrichment [70]. In a study by Lampignano et al. initiated by the CANCER-ID/ELBS consortium [73] different technologies for ctDNA purification, quantification, and characterization were evaluated in a multi-center study. Using spike-in experiments of TP53 mutated lung cancer cell line DNA in healthy donor blood, the study demonstrated the feasibility of mutation detection by NGS and ddPCR independent of the laboratory [74].

 Despite these challenges, several liquid biopsy tests have already found their way into clinical practice.

 The presence of CTCs has been shown to be a negative prognostic marker for overall survival and can be used to identify those patients at risk for relapse or progression [75]. Their clinical importance of CTC detection was acknowledged by the American Joint Committee’s (AJCC). Their breast cancer staging manual was updated in 2018 and includes the a new classification, cM0(i+), which is used to describe the presence of CTCs and or DTC clusters (≤ 0.2 mm) in the bone marrow or other organs distant from regional lymph nodes and the breast [76].

 Moreover, the detection and genomic analysis of ctDNA has been proven to be a valuable tool in personalized medicine [77], especially with the recent advances in targeted therapy as well as immune checkpoint inhibition (ICI). The European Society for Medical Oncology (ESMO) therefore issued recommendations on the usage of ctDNA based assays for genotyping and treatment selection due to their high validity and clinical utility [78]. The detection of targetable mutations, e.g. *EGFR* in NSCLC or *KRAS/NRAS/BRAF* V600E in colorectal cancers, could lead to a faster diagnosis and treatment selection and could be taken into account when tissue biopsies are not available. Taken together, these recent advances have led to the implementation of several tests into the clinical routine including the analysis of targetable *EGFR* mutations and *ALK* rearrangements in NSCLC ctDNA [79], analysis of AR-V7 status in CTCs from mCRPC patients [80], and also multigene assays such as the NGS-based Guardant360 CDx [81]. Recent studies have also shown clinical utility of CTC enumeration and characterization for therapeutic decision making in metastatic breast cancer [82, 83].

 However, the possibility of false-positive findings needs to be considered which might derive from clonal expansion of apoptotic hematopoietic cells in the blood (clonal hematopoiesis of indeterminate potential (CHIP)) [84]. False-negative results could furthermore occur when only low levels of plasma DNA are available, or the tumor does not shed sufficient DNA [78]. To address these challenges for the detection of MRD in colorectal, pancreatic and lung cancer the EU/IHI-funded GUIDE.MRD consortium was initiated in 2023 [85, 86].

## Conclusions

 In conclusion, there are still hurdles that need to be addressed before liquid biopsy tests will find their way into routine clinical practice. High costs for detection devices and personnel as well as their training, and the constant need for tumor surveillance due to its spatial and temporal heterogeneity are examples for the challenges that are faced today.

 To overcome these obstacles, several international projects such as CANCER-ID (2015–2019), which was carried out under the umbrella of the European Union Innovative Medicines Initiative (IMI), have been established to standardize protocols for clinical validation of liquid biopsy methods. Apart from that, EU-wide as well as internationally active consortia and networks such as the European Liquid Biopsy Society (ELBS) provide a network of academic and industry partners which aim at closing the gap between the multitude of scientific publications and clinical implementation of liquid biopsy [87]. Moreover, international efforts are currently being made to update the RECIST criteria (Response Evaluation Criteria In Solid Tumors) by including ctDNA analysis for treatment response evaluation in solid tumors [88].

 Despite the many challenges that still need to be overcome, the current advances in research and the joint efforts of academia and industry partners pave the way for the establishment of a successful clinical implementation of liquid biopsy. These endeavours will ultimately change cancer treatment through improvements in early detection, risk assessment and patient monitoring to prolong survival rates and enhance quality of life of many patients in the future.

## Abbreviations

AJCC: American Joint Committee
CAF: Cancer-associated fibroblast
CDX: CTC-derived eXplant
CEC: Circulating endothelial cell
CHIP: Clonal hematopoiesis of indeterminate potential
CNA: Copy number alteration
CSC: Cancer stem cell
CTC: Circulating tumor cell
DTC: Disseminated tumor cell
ELBS: European Liquid Biopsy Society
EMT: Epithelial–mesenchymal transition
EpCAM: Epithelial cell adhesion molecule
EPISPOT: Epithelial ImmunoSPOT
ER: Estrogen receptor
ESMO: European Society for Medical Oncology
EV: Extracellular vesicle
FDA: Food and Drug Administration
ICI: Immune checkpoint inhibition
ILSA: International Liquid Biopsy Standardization Alliance
IMI: Innovative Medicines Initiative
NGS: Next generation sequencing
NSCLC: Non-small cell lung cancer
MET: Mesenchymal-epithelial transition
PDX: Patient-derived xenograft
RECIST: Response Evaluation Criteria In Solid Tumors
tdEVs: Tumor-derived EVs
WGA: Whole genome amplification
